# Fading into Obscurity: Impact of Climate Change on Suitable Habitats for Two Lesser-Known Giant Flying Squirrels (Sciuridae: *Petaurista*) in Northeastern India

**DOI:** 10.3390/biology14030242

**Published:** 2025-02-27

**Authors:** Imon Abedin, Manokaran Kamalakannan, Tanoy Mukherjee, Anwaruddin Choudhury, Hilloljyoti Singha, Joynal Abedin, Dhriti Banerjee, Hyun-Woo Kim, Shantanu Kundu

**Affiliations:** 1 Department of Zoology, Bodoland University, Kokrajhar 783370, India; 2 Mammal and Osteology Section, Zoological Survey of India, Kolkata 700053, India; 3 Agricultural and Ecological Research Unit, Indian Statistical Institute, Kolkata 700108, India; 4 The Rhino Foundation for Nature in NE India, Guwahati 781007, India; 5 Centre for Wildlife Research and Biodiversity Conservation, Bodoland University, Kokrajhar 783370, India; 6 Dibru-Saikhowa Conservation Society, Tinsukia 786147, India; 7 Zoological Survey of India, Prani Vigyan Bhawan, Kolkata 700053, India; 8 Department of Marine Biology, Pukyong National University, Busan 48513, Republic of Korea; 9 Marine Integrated Biomedical Technology Center, National Key Research Institutes in Universities, Pukyong National University, Busan 48513, Republic of Korea; 10 Department of Biology, Faculty of Science and Technology, Airlangga University, Surabaya 60115, Indonesia; 11 Ocean and Fisheries Development International Cooperation Institute, College of Fisheries Science, Pukyong National University, Busan 48513, Republic of Korea; 12 International Graduate Program of Fisheries Science, Pukyong National University, Busan 48513, Republic of Korea

**Keywords:** eastern Himalaya, small mammals, endemic species, species distribution modeling, habitat, conservation biology

## Abstract

In recent years, global warming has become a major driver of biodiversity loss, significantly impacting various vertebrate species, including mammals. Consequently, numerous smaller species face extinction risks due to anthropogenic factors as well as inadequate assessments and conservation planning. Thus, this study focuses on two recently described endemic giant flying squirrel species under the *Petaurista* genus—*Petaurista mishmiensis* and *Petaurista mechukaensis*—found in Arunachal Pradesh, India. Using an ensemble species distribution model (SDM), this research delineates suitable habitats for these lesser-known species and evaluates the effects of climate change and habitat fragmentation on these areas. This analysis aims to inform a comprehensive management plan for their conservation. The ensemble model identified suitable habitat patches for the two species, extending beyond their current IUCN-designated ranges in Arunachal Pradesh. Under present conditions, *P. mishmiensis* has the largest predicted suitable area (9213 sq. km), followed by *P. mechukaensis* (6754 sq. km). However, future projections reveal alarming habitat losses ranging from 13.45% to 55.86% across the study area. This study also highlights severe habitat fragmentation throughout the state as viable patches for *P. mishmiensis* are drastically reduced in size, resulting in many being completely lost and the remaining areas being closer together. However, the *P. mechukaensis* experiences significant disintegration, resulting in numerous smaller, more dispersed patches within Arunachal Pradesh. Hence, to address these challenges, this study recommends several actions such as genetic assessments to confirm evolutionary relationships, evaluations of corridor connectivity, and comprehensive field studies. Furthermore, establishing joint forest conservation committees involving local communities, forest personnel, defense forces, naturalists, and scientists are also encouraged. Ultimately, this research provides critical insights for guiding future field studies across Arunachal Pradesh’s vast landscapes and supports the development of detailed species management plans to protect these endemic flying squirrels.

## Simple Summary

In recent decades, two new species of giant flying squirrels (genus *Petaurista*) have been described in Arunachal Pradesh, India, occupying small, isolated patches between major river systems. However, there has been no comprehensive assessment of these species, and no conservation plans have been developed to safeguard them. This study aims to delineate suitable habitats for these species and evaluate the potential impacts of future climate change on these areas. The results reveal alarming trends, with both species projected to experience significant declines in their suitable habitat ranges. Additionally, shifting climatic conditions are expected to cause severe habitat fragmentation within their ranges. Hence, to mitigate these challenges, this study advocates for extensive field research, genetic assessments, corridor connectivity evaluations, and the formation of joint conservation committees to develop a comprehensive species management strategy.

## 1. Introduction

In recent years, global warming has emerged as a significant driver of biodiversity loss, profoundly affecting a wide range of vertebrate species, including mammals [1,2]. This phenomenon has accelerated species decline and, in some cases, driven them to extinction within their ecological niches [3,4,5]. Alarmingly, current extinction rates are estimated to be approximately 1000 times higher than historical background levels, with projections indicating a continued increase in the coming decades [6]. This trend is primarily attributed to the destruction of native habitats, which not only disrupts individual fitness and functional diversity but also leads to significant changes in species richness and community structure [7]. These native niches play a crucial role in species survival by facilitating interactions with environmental conditions and ensuring the long-term persistence of viable populations [8]. Specifically, urbanization is widely recognized as a major threat to vertebrate species, and with the pace of urban expansion accelerating globally, there is an urgent need to prioritize research and conservation efforts aimed at safeguarding species that inhabit human-modified environments [8,9,10]. While numerous studies have been conducted to address these challenges, the majority have predominantly focused on charismatic species and have left many less conspicuous species understudied and poorly understood [11,12,13,14]. This disparity in attention is especially concerning because effective conservation management relies heavily on scientific research work. Such information is critical for optimizing the allocation of limited conservation resources and implementing legislative tools to protect biodiversity. One of the most effective ways to bridge this knowledge gap is by predicting species distributions and identifying the underlying drivers of spatial patterns and population dynamics [4]. This can aid in enhanced understanding of species ecology and improve conservation outcomes in an increasingly urbanized world.

Flying squirrels (Sciuridae: Rodentia) have received limited and sporadic attention in scientific studies despite their ecological importance [15]. They are vital for forest ecosystem functioning, providing critical services like pollination, seed dispersal, etc. and act as indicators of ecosystem health [16,17]. These mammals are unique due to the presence of a membrane or “parachute” that stretches between their limbs and enables them to glide. A total of 37 species of flying squirrels have been recognized globally under eight genera: *Belomys*, *Biswamoyopterus, Eoglaucomys, Eupetaurus, Hylopetes, Petaurillus, Petaurista*, and *Petinomys* [18,19]. The evolution of their gliding ability is believed to be a natural extension of their arboreal lifestyle and signifies an adaptive progression within forested environments [20,21]. This specialized locomotion has likely contributed to the diversification of the vertebrate lineages that developed it by allowing them to access tall canopies, exploit resources more efficiently, and evade predators, thus opening up numerous ecological opportunities [22,23]. Despite their importance, the population of flying squirrels has been declining over the past few decades mainly due to habitat loss caused by deforestation and the degradation of primary forests, along with hunting pressures in regions like India [24,25,26,27,28]. Almost all species of flying squirrels are confined to Asia, with most distributed across Southeast Asia, hence it is recognized as a hotspot for flying squirrels [15]. In India, there are 23 recorded species of flying squirrels, with 18 of them recorded from Northeast India, a region characterized by their restricted ranges and endemic populations of biodiversity [29]. Notably, two species of the *Petaurista* genera viz. *Petaurista mishmiensis* and *Petaurista mechukaensis* have been identified based on holotypes collected exclusively from hunters in Arunachal Pradesh and were submitted to the National Zoological Collections of Zoological Survey of India [30,31,32]. Though there is some debate regarding the taxonomy of these species, the IUCN-SSC Small Mammal Specialist Group (SMSG) has assessed and included them in the IUCN Red List of Threatened Species, formally recognizing them as distinct species [33,34]. Moreover, they have classified *P. mishmiensis* and *P. mechukaensis* as ‘Near Threatened’ having very localized distributions, confined to Mechuka and Mishmi Hills/Dibang Valley to Anjaw in Arunachal Pradesh, India. Their limited range, coupled with rapid deforestation in these areas, poses a significant threat to their survival. Furthermore, to date, only a single ecological modeling and habitat suitability study on *Petaurista philippensis* has been conducted within the Indian subcontinent [35]. Hence, there is a significant gap in understanding the habitat preferences of *Petaurista* flying squirrels and their response to climate change in the Asian context, which is crucial for developing an effective conservation management plan. This is particularly important as climate change is expected to exert substantial pressure on many small taxa, further increasing their vulnerability [36].

In this regard, to evaluate the vulnerability of taxa to climate change, species distribution modeling (SDM) offers valuable insights [37,38,39,40]. The SDM has proven indispensable for uncovering ecological and biogeographical relationships, which form the basis for designing and implementing targeted conservation and management strategies [41,42]. Among the various SDM approaches, ensemble modeling has emerged as a powerful tool for estimating habitat suitability across species. This method integrates multiple modeling algorithms to predict species distributions, effectively capturing the diverse factors and underlying processes influencing their geographic range [43]. Thus, by balancing the strengths and weaknesses of individual models, ensemble approaches enhance prediction accuracy and reliability, making them particularly effective for conservation planning. Moreover, understanding how key environmental variables respond to climate change is critical for identifying suitable habitats. This knowledge is vital for developing effective conservation strategies and landscape-level management plans [44,45]. Accordingly, the present study employs an ensemble SDM to delineate the suitable habitats of two lesser-known *Petaurista* giant flying squirrel species in Arunachal Pradesh and evaluate the impacts of climate change and fragmentation on these suitable areas. This analysis aims to inform and support the formulation of a comprehensive management plan for the collective conservation of these locally distributed species.

## 2. Materials and Methods

### 2.1. Study Area

**Figure 1:**
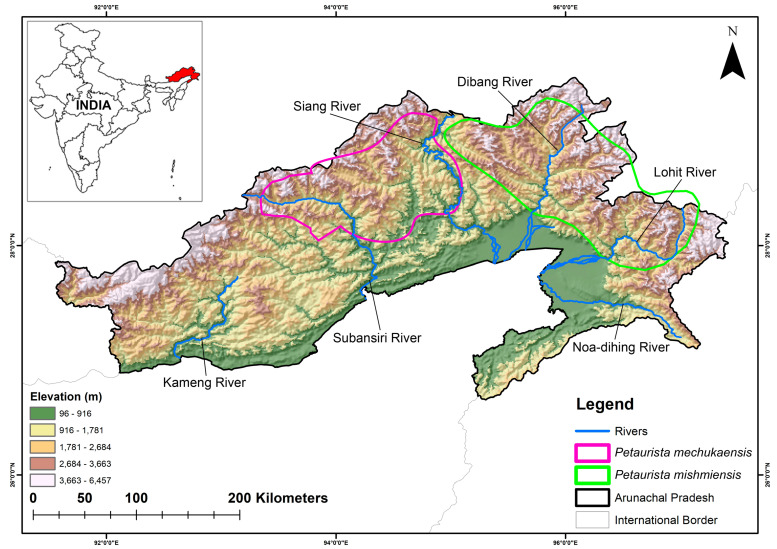
Map of the study area, i.e., Arunachal Pradesh, India, showing the IUCN-designated range of the two targeted *Petaurista* flying squirrel species. The elevation and major riverine systems within the region are also highlighted.

The type specimens of *P. mishmiensis* and *P. mechukaensis* were collected from Arunachal Pradesh, India, and have been deposited in the National Zoological Collections of the Mammal & Osteology Section, Zoological Survey of India, Kolkata. Morphologically, *P. mishmiensis* exhibits a head and body length ranging from 405 to 590 mm, a tail between 570 and 600 mm, a dorsum that varies from deep chestnut to blackish chestnut, an orange-buff underside, deep chestnut patagium, a black tail with the basal one-third deep chestnut, and black feet. However, *P. mechukaensis* has a head and body length of 460–530 mm, a tail length of 520–770 mm, a dorsum that appears deep chestnut-black, an orange-buff venter, a black tail with the basal half deep gray, and black feet [30,31,32]. In particular, the type specimen of *P. mishmiensis* was obtained from the Mishmi Hills within the Dibang Valley, alongside the Dibang River, whereas *P. mechukaensis* has been obtained from two sites: Mechuka Valley and the mountainous region connecting Mechuka and Tato [30]. These both sites were located in Shi Yomi District, which was formerly part of the West Siang District [31] (Figure 1). Hence, to corroborate their localities and distinct geographic distributions, the entire state of Arunachal Pradesh was used as the training extent for this study. This approach was also intended to identify potential climate refugia that could be designated as protection sites and to explore the factors that may have contributed to the diversification of these species. This study primarily focused on the opportunistic sightings for two species, i.e., *P. mishmiensis* and *P. mechukaensis*, in the Mechuka to Tato area and the Mishmi Hills (covering areas such as Tiwari Gaon, Hunli, Etalin, and a 65 km stretch in Anjaw, respectively) (Figure 1). Using this approach, several presence locations (*P. mishmiensis* = 34 and *P. mechukaensis* = 21) were identified, which were subsequently used for further analysis in this study. The spatial correlation of occurrence data was analyzed at a resolution of 1 km^2^ using SDM Toolbox v2.4 [46]. This resolution was chosen to match the pixel size of the raster data, thereby minimizing overfitting and improving model accuracy. Hence, the final model was run using 31 points for *P. mishmiensis* and 19 points for *P. mechukaensis.*

### 2.2. Habitat Requirements

These two flying squirrel species predominantly inhabit the montane forests of Arunachal Pradesh, with distinct elevation preferences. Specifically, *P. mishmiensis* is primarily found at elevations ranging from 600 to 1600 m above sea level (msl), whereas *P. mechukaensis* occurs at higher altitudes, between 1500 and 2500 msl. Furthermore, their distributions are further segregated by major rivers, including the Subansiri, Siang, Dibang, and Lohit. Moreover, the climate of this state also varies significantly with topography and elevation [47]. The foothill zone has a subtropical climate characterized by hot and humid conditions. In the lower valleys, summer temperatures (June–August) often reach the mid-90s °F (mid-30s °C), while winter high temperatures (December–February) typically remain around the mid-50s °F (approximately 13 °C). The temperature gradually decreases with increasing elevation in the mountainous regions. Additionally, the state experiences a distinct wet–dry monsoon pattern, influencing overall precipitation levels.

### 2.3. Selection of Covariates

To identify suitable habitat patches for the flying squirrels in the study area, a combination of bioclimatic, topographic, habitat, and anthropogenic variables was utilized [42]. The standard set of 19 bioclimatic variables was obtained from the WorldClim database (https://www.worldclim.org/, accessed on 13 November 2024) and extracted for use within the study area [48]. Since these species primarily occur in montane forests, the Euclidean distance to montane forest was selected as a habitat variable. This raster was derived from the Land Use and Land Cover data of Copernicus and was processed using the Euclidean distance function in ArcGIS v. 10.8 [49]. Additionally, because the species are distributed between riverine systems, the Euclidean distance to rivers was also incorporated as a variable. Furthermore, the topographic variables, such as elevation, aspect, and slope, were extracted using 90 m Shuttle Radar Topography Mission (SRTM) data (http://srtm.csi.cgiar.org/srtmdata/, accessed on 13 November 2024). The Global Human Footprint Dataset was utilized as an anthropogenic predictor to assess the Human Influence Index (HII) and understand the extent of human impact on the target species [50]. All spatial variables were standardized to a resolution of 30 arcseconds (~1 km^2^) using the spatial analyst extension in ArcGIS 10.6. Moreover, to ensure robustness in the analysis, spatial multicollinearity testing was performed using the SAHM (Software for Assisted Habitat Modeling) package in VisTrails software [51]. The variables with a Pearson correlation coefficient (r) exceeding 0.8 were excluded from further analysis to minimize redundancy [52]. Moreover, the final dataset of predictor variables included 11 key variables used for modeling the habitat suitability of the two giant flying squirrel species (Figures S1 and S2).

Furthermore, to assess the impacts of climate change, this study analyzed future climate scenarios under two Shared Socioeconomic Pathways (SSPs): SSP245 and SSP585, for the periods 2041–2060 and 2061–2080. The future climate projections were based on the HadGEM3-GC31 LL model that is part of the Coupled Model Intercomparison Project Phase 6 (CMIP6). This model was chosen for its reliability in simulating climate variability and temperature trends across South and Southeast Asia [53,54,55]. Additionally, to focus exclusively on the effects of climate change on species distribution, non-climatic variables were held constant during future climate analyses as this approach ensured that the projections remained in ecologically relevant areas [39,56].

### 2.4. Model Building and Evaluation

The habitat modeling in this study employed the ensemble approach that integrated multiple algorithms to construct a comprehensive model for the target species. This ensemble approach leverages the strengths of various algorithms to predict suitable habitats across geographic regions, capturing the diverse factors that influence species distribution [43]. Thus, by combining the advantages and mitigating the limitations of individual models, this integrated method improves the accuracy and reliability of distribution predictions. The four distinct algorithms chosen for this study were Multivariate Adaptive Regression Splines (MARS), Generalized Linear Model (GLM), Maximum Entropy (MaxEnt), and Random Forest (RF) [57,58,59]. These models were implemented using the SAHM package in VisTrails software, which produced probability maps with values ranging from 0 (least suitable) to 1 (most suitable).

Additionally, binary maps were generated by applying the minimum training presence as the threshold [51,60]. The model performance was evaluated using the area under the curve (AUC) metric, with a threshold of 0.75 set as the primary criterion for model validation [61,62]. To assess habitat configurations for the species, an ensemble count map was generated, where each pixel represented the agreement among the different models. In addition to assessing the performance of the models, a few more robust performance metrics, such as the ΔAUC, true skill statistic (TSS), Cohen’s Kappa, Proportion Correctly Classified (PCC), specificity, and sensitivity, were calculated for both the training and cross-validation datasets (*n* = 10). Moreover, the mean contribution percentage of each variable was determined by averaging its contribution across all models. These metrics ensured the reliability and robustness of the final model in predicting species distribution [63,64,65,66].

### 2.5. Assessment of Habitat Quality

The qualitative and geometric characteristics of suitable habitat patches for the two flying squirrel species were assessed under both current and projected future climatic scenarios to facilitate comparative analyses. To conduct this evaluation, this study utilized class-level metrics with FRAGSTATS software version 4.2.1 [67]. This widely recognized software in landscape ecology and environmental management allows for the analysis of spatial patterns within habitats, offering a comprehensive suite of metrics and indices to assess and clarify the structure and composition of landscapes [68]. The analysis incorporated several key metrics for shape geometry analysis, such as the number of patches (NP), largest patch index (LPI), aggregate index (AI), patch density (PD), total edge (TE), and landscape shape index (LSI). The metrics such as NP, PD, TE, and LPI provide detailed insights into the shape and geometry of suitable habitat patches, examining their size, edge characteristics, and density within a defined geographical area. In contrast, the LSI metric evaluates the complexity of patch shapes, while the AI quantifies the proximity or clustering of patches, reflecting the degree to which they are aggregated or dispersed across the landscape. These metrics collectively contribute to a comprehensive understanding of habitat structure, essential for assessing the viability of these habitats under both current and future climate conditions.

## 3. Results

### 3.1. Evaluation of Models and Variable Importance

**Figure 2:**
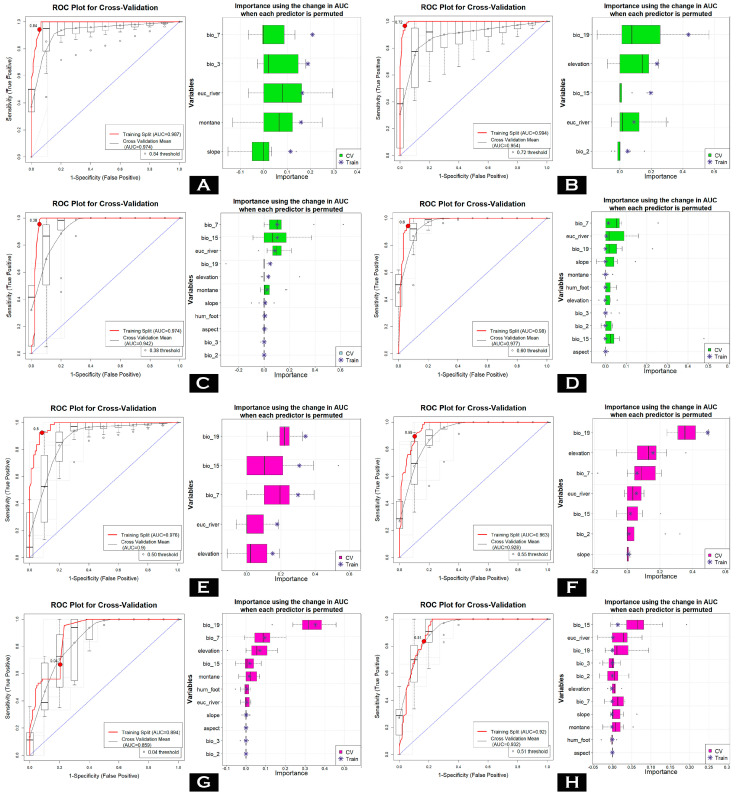
Model evaluation plots depicting the average training ROC for both training and cross-validation (CV), along with the predictors selected by the model across replicate runs under four different models. Generalized Linear Model (GLM) of (**A**) *P. mishmiensis* and (**E**) *P. mechukaensis*; Multivariate Adaptive Regression Splines (MARS) of (**B**) *P. mishmiensis* and (**F**) *P. mechukaensis*; Maximum Entropy (MaxEnt) model for (**C**) *P. mishmiensis* and (**G**) *P. mechukaensis*; Random Forest (RF) model for (**D**) *P. mishmiensis* and (**H**) *P. mechukaensis*.

The ensemble model analysis across four modeling approaches for the two flying squirrels yielded robust results, with AUC values consistently exceeding the threshold of 0.75 (Figure 2, Table 1). These high AUC values were maintained in both the training and cross-validation phases for both species, indicating strong model performance. Among the modeling approaches, MARS exhibited the highest ΔAUC values for *P. mishmiensis*, whereas the GLM model achieved the highest ΔAUC for *P. mechukaensis*. In contrast, the RF model produced the lowest ΔAUC values across both species (Figure 2, Figures S3 and S4 and Table 1). In addition to AUC and ΔAUC, other evaluation metrics such as PCC, TSS, kappa, specificity, and sensitivity also demonstrated high values, further confirming the robustness and reliability of the models in predicting suitable habitats for these species.

The model identified the Precipitation of the Coldest Quarter (bio_19) as the most influential variable contributing to habitat suitability for the two flying squirrel species in Arunachal Pradesh (Table 2, Figure 2). This variable accounted for 42.58% for *P. mechukaensis* and 21.34% for *P. mishmiensis*. Furthermore, the topographic variable elevation also emerged as a significant predictor for both species as the highest contribution among the studied species was observed for *P. mechukaensis* at 15.27%, while the lowest was noted for *P. mishmiensis*, contributing 11.83%. Moreover, the habitat variable Euclidean distance to waterbodies (euc_river) was another critical factor delineating suitability. It contributed 8.79% for *P. mechukaensis* and 15.05% for *P. mishmiensis*. Additionally, the anthropogenic factor, i.e., the Human Influence Index (Human_foot), also played a role and contributed 0.11% to *P. mechukaensis* and 0.26% to *P. mishmiensis*.

### 3.2. Habitat Suitability in Present and Future Climate Scenarios

**Figure 3:**
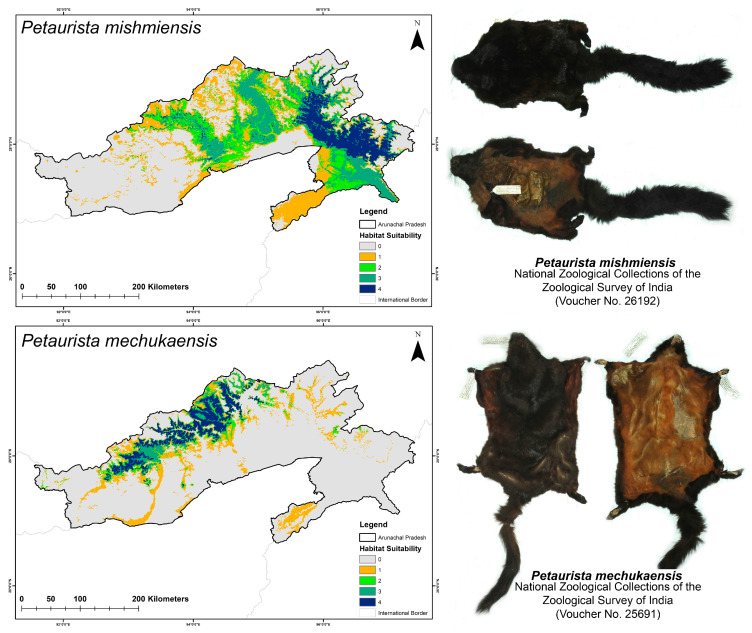
Maps depicting the suitable habitats for the two *Petaurista* flying squirrel species identified by the model under the present climatic scenario in Arunachal Pradesh, India. The different colors and numbers represent the level of model agreement, with “0” indicating no model agreement and “4” signifying high suitability where all four models concurred. Additionally, photographs of museum specimens archived at the National Zoological Collections of the Mammal & Osteology Section, Zoological Survey of India are provided along with their corresponding voucher numbers.

Within the study area, the analysis revealed that the species *P. mishmiensis* had the largest extent of suitable habitat, spanning 9213 sq. km, followed by *P. mechukaensis*, with a suitable area of 6754 sq. km under current conditions (Figure 3, Table S1). Furthermore, when evaluating the suitability within the IUCN-designated ranges for these species, *P. mechukaensis* exhibited the lowest proportion of suitable habitat within its IUCN-designated range, accounting for only 28.08%, whereas for *P. mishmiensis*, 33.46% of its IUCN-designated extent was found to be suitable under the present conditions (Table S1).

**Figure 4:**
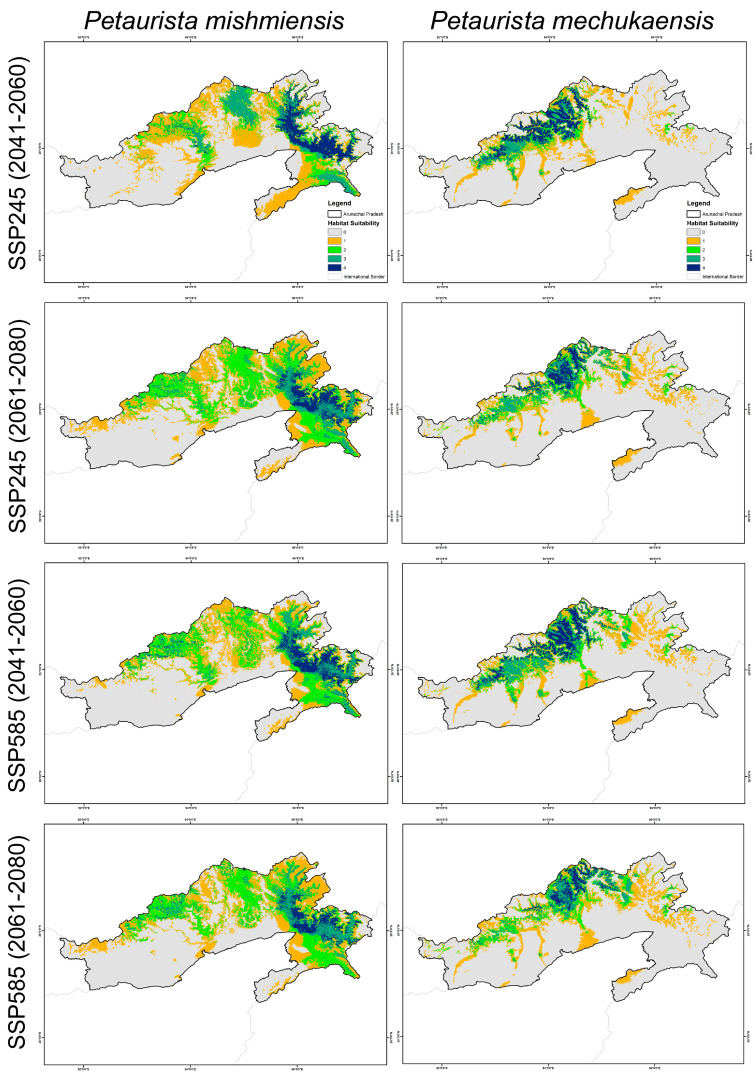
Maps depicting the suitable habitats for the *Petaurista* flying squirrel species identified by the model under various future climatic scenarios in Arunachal Pradesh, India. The different colors and numbers represent the level of model agreement, with “0” indicating no model agreement and “4” signifying high suitability where all four models concurred. The columns represent the species, while the rows indicate the different SSP scenarios and timeframes.

**Figure 5:**
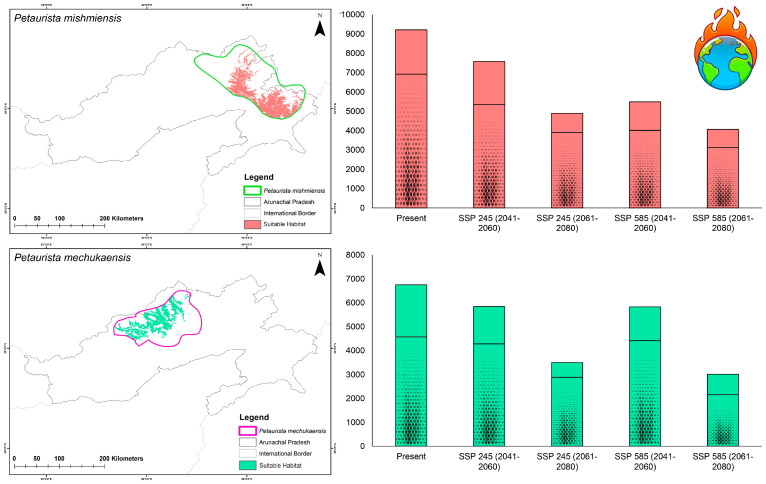
Maps illustrating the suitable habitats within the IUCN-designated range for the *Petaurista* flying squirrel species under the present climatic scenario. The accompanying bar graph represents the total suitable area for each species within Arunachal Pradesh, India, with the patterns on the bars indicating the proportion of suitable habitat that falls within the IUCN-range.

Under future climate scenarios, both the lesser-known flying squirrel species are projected to experience a significant decline in habitat suitability within the training extent as well as their designated IUCN extents (Figure 4 and Figure 5). However, under future climatic scenarios, both *P. mishmiensis* and *P. mechukaensis* are projected to experience a decline in habitat suitability. Specifically, the habitat suitability for *P. mishmiensis* is projected to decline by 17.72% to 55.86%, while *P. mechukaensis* is expected to experience a decline ranging from 13.45% to 55.43% across Arunachal Pradesh in future scenarios (Figure 4 and Figure 5, Table S1). However, contrasting patterns emerge within their IUCN-designated extents in the future due to climatic shifts. Specifically, for *P. mechukaensis*, the decline in suitable habitat within its IUCN extent is relatively moderate, ranging from 6.40% to 52.72%, with most losses occurring outside its designated range. In contrast, *P. mishmiensis* shows higher losses within its IUCN-designated extent, with habitat suitability declining by 22.75% to 54.86% (Table S1). Across both species, the SSP245 scenario is associated with relatively lower habitat losses compared to SSP585 in both future timeframes.

### 3.3. Assessment of Habitat Shape Geometry Dynamics

Climate change in the future is expected to result in significant changes in habitat dynamics and lead to increased fragmentation and alterations in patch geometry for the two studied species in Arunachal Pradesh (Table 3). These changes are particularly pronounced for *P. mishmiensis* across Arunachal Pradesh as it faces significant challenges due to the loss and alteration of viable habitat patches. The NP is projected to decrease by up to 16.99%, and patch sizes are expected to shrink significantly with the LPI declining by 79.73% (Table 3). The reduction in patch edges is evident as TE decreases by over 27%. Moreover, the patches for *P. mishmiensis* are expected to exhibit more complex geometries in the future, as indicated by a 20% increase in the LSI. Moreover, the fragmentation is also apparent for *P. mishmiensis*, with increased separation between habitat patches as reflected by a decline in the AI of up to 18.94% under future climatic scenarios.

Furthermore, *P. mechukaensis* is projected to experience significant habitat fragmentation in the future due to climate change. Unlike *P. mishmiensis*, where viable patches are largely lost, *P. mechukaensis* exhibits fragmentation characterized by the disintegration of larger patches into many smaller ones (Table 3). This is reflected in an increase in the NP and PD by over 28.94% and 28%, respectively, alongside a decline in the LPI and TE by up to 81.64% and 28.20%, respectively. Moreover, the fragmentation is further indicated by reduced proximity between patches, with the AI showing a decline of up to 14.84% in future scenarios. Collectively, these changes highlight severe fragmentation for *P. mechukaensis*, with larger patches breaking into numerous smaller, more isolated patches. In contrast, *P. mishmiensis* is projected to lose many viable patches entirely, leaving smaller, distant patches that contribute to reduced connectivity across their habitat ranges.

## 4. Discussion

The world is currently experiencing the sixth mass extinction as the species extinction rates are significantly higher than the natural baselines [69]. In this critical scenario, small-ranged and island species are particularly vulnerable to extinction compared to widely distributed species due to their limited range, smaller population sizes, and increased susceptibility to stochastic events [70]. This heightened susceptibility is largely attributed to ongoing climatic shifts driven by anthropogenic activities, which underscores the urgent need for effective biodiversity conservation strategies [71]. Thus, to address these challenges, it is imperative to implement targeted species- and site-specific conservation measures to prevent the extinction of such vulnerable species [69]. Considering these threats, it has become increasingly important to assess lesser-known species inhabiting the northeastern forests as this region remains significantly understudied [72]. Hence, this study focuses on evaluating the critical impacts of climate change and habitat dynamics on two lesser-known, small-ranged flying squirrel species that have only been reported from Arunachal Pradesh, India. These findings are expected to provide essential insights, aiding in rigorous field surveys and the development of effective conservation and management plans for these species and other sympatric species within this biodiversity-rich region.

The ensemble model identified suitable habitat patches for the two flying squirrel species both within and beyond their IUCN-designated extents in Arunachal Pradesh. Under the current scenario, the model predicted the largest suitable area for *P. mishmiensis* (9213 sq. km), followed by *P. mechukaensis* (6754 sq. km). Specifically, for *P. mishmiensis*, the model identified suitable areas primarily along the Dibang River, encompassing the Mishmi Hills and extending up to the Anjaw district north of the Lohit River [31,34]. Hence, the areas especially from Anini, Mayudia, Udayak Pass, Walong, Kaho, etc., shall be rigorously field validated to better understand their occupancy in this region. Moreover, for *P. mechukaensis*, the model predicted suitable habitats between the Siang and Subansiri Rivers extending further westward up to the Kameng River. While the designated range of this species lies east of the Subansiri River and west of the Siang River, the suitable areas identified beyond this area up to the Kameng River aligns with the potential range of this species [32,33].

Furthermore, the present study identified the bioclimatic variables related to precipitation and temperature, such as Precipitation of the Coldest Quarter (bio_19) and Temperature Annual Range (bio_7) as significant factors influencing the distribution of the two flying squirrel species in Arunachal Pradesh. These findings are aligned with previous studies that highlight the importance of these bioclimatic variables as important determinants for flying squirrel distribution across their range [73,74,75]. Moreover, these variables are also crucial for the forests in higher elevation areas, which could significantly influence the distribution patterns of these faunal species [75,76]. Additionally, the Euclidean distance to rivers (euc_river) was a highly predictive variable for both species, aligning with their habitat preferences and affinity for riverine systems [33,34]. This variable likely explains the segregation of these species, as the major river systems in Arunachal Pradesh may act as natural barriers, restricting their ranges to specific areas between these riverine systems. Furthermore, elevation also emerged as a key factor influencing their distribution, as it contributed 15.27% for *P. mechukaensis* compared to 11.83% for *P. mishmiensis*. This finding aligns with the known habitat preference of *P. mechukaensis* for higher elevations, distinguishing it from *P. mishmiensis*. The anthropogenic variable Human Influence Index had a minimal impact on both species, contributing only 0.26% and 0.11% for *P. mishmiensis* and *P. mechukaensis*, respectively. This may be attributed to Arunachal Pradesh having the lowest human population density in India, largely due to its rugged terrain, harsh climate, and dense forests. Additionally, the recent initiation of large-scale development projects could impact wildlife habitats, including those of these two flying squirrel species, in the near future.

However, climate change has significantly impacted these two species, as the habitat loss is projected to range from 13.45% to 55.86% across the entire training extent in future scenarios. This observation aligns with studies indicating that high-elevation species are particularly vulnerable to climatic shifts, as demonstrated by the decline in habitat suitability for both species under future projections [77,78,79]. The reduction in suitable areas for both the giant flying squirrels is concerning given that they already occupy a small, isolated extent. This finding corroborates previous research showing that species with small, restricted ranges are more susceptible to climate change, which further increases their extinction risk [80,81,82]. This climate-induced loss of suitable areas in future scenarios has also resulted in severe habitat fragmentation across the ranges of these species. This fragmentation is evident from the assessment of the shape geometry of suitable habitat patches under different climatic projections. Specifically in the situation for *P. mishmiensis*, many viable patches are completely lost, and the remaining patches are significantly reduced in size with increased proximity among them. In contrast, for *P. mechukaensis*, many viable patches become disintegrated, thus forming numerous smaller patches that are more widely dispersed. These remaining small patches under future climatic scenarios are critical for the survival of these endemic flying squirrels and warrant further study with targeted conservation plans that must be implemented to ensure their persistence [83]. The survival of these species depends on their ability to persist within the remaining fragmented patches, as failure to do so could result in their extinction [83,84].

Thus, to ensure the conservation of these endemic flying squirrels, rigorous field assessments across various regions of Arunachal Pradesh are imperative. These assessments will validate the findings of the present study and provide critical insights into the species’ ecology, which will form the foundation for drafting effective species management plans. It is further recommended to conduct molecular studies to delineate and confirm the taxonomic status of these squirrels and their type localities. While the IUCN-SSC SMSG has recognized them based on morphological characteristics, genetic analyses will provide phylogenetic insights, establish evolutionary timelines, and assess whether riverine systems acted as barriers to their dispersal and led to speciation. Additionally, corridor connectivity assessments are essential to evaluate the isolated ranges of these species. The field expeditions should also be conducted in the suitable areas delineated by the current study to assess habitat viability and investigate the potential presence of other similar species. The other recently described species, such as *Biswamoyopterus biswasi* and *P. siangensis*, also require thorough field assessments to determine their distribution and ecological characteristics [85,86,87]. These evaluations are essential for assessing the impact of climate change, facilitating SDM studies to evaluate habitat suitability, and projecting future climatic effects to prioritize conservation efforts [88]. Furthermore, the contiguous forest patches near Mechuka and other regions of the Mishmi Hills, Walong, Wakro, Hawai, Udayak Pass, etc., require special attention, including the designation of protected area status. This can be achieved through dialogues with local communities to ensure their active participation. Moreover, the forest department should be facilitated by other governmental agencies, non-governmental organizations, IUCN-SSC SMSG and research institutions for comprehensive assessments and implementation of conservation strategies. Furthermore, awareness campaigns among local tribal communities are essential, as traditional hunting practices targeting small mammals pose a significant threat to these species. These campaigns must be conducted sensitively to respect cultural practices while promoting conservation. Moreover, it is imperative to conduct comprehensive environmental impact assessments (EIAs) for any developmental projects undertaken in these ecologically fragile landscapes. Additionally, addressing land conversion issues, such as the establishment of palm oil plantations in the forested areas of the Mishmi Hills, is crucial for the conservation of flying squirrels. The formation of joint forest conservation committees involving village leaders, forest personnel, defense personnel, naturalists, scientists, and other stakeholders is recommended. These committees will play a pivotal role in monitoring, facilitating conservation activities, and fostering community-driven initiatives. Finally, safeguarding both current and future suitable habitat patches is vital to mitigate extinction risks. By addressing these priorities, the present study provides valuable information to guide field surveys and aid in the formulation of robust species management plans for the conservation of these unique flying squirrels.

## 5. Conclusions

The northeastern states of India are home to a rich biodiversity, hosting numerous endemic species. In recent decades, two new species of flying squirrels have been reported from very restricted areas within this region. The present study applied SDM to delineate their suitable habitats and project their future suitability under changing climatic conditions. The findings highlight concerning trends, as both species experience a significant decline in suitable habitat due to climate change. The climatic parameters that most strongly influence their distribution were identified, with a clear affinity for water bodies and montane forests. Given these findings, several recommendations are made, including the need for genetic assessments to confirm their evolutionary biology, the evaluation of corridor connectivity, and the implementation of rigorous field studies. Additionally, the formation of joint forest conservation committees involving local communities, forest personnel, defense personnel, naturalists, and scientists is encouraged. Moreover, support for the forest department from various government line departments, NGOs, and other agencies is also emphasized. Ultimately, this study provides valuable information for conducting further field research across the expansive landscapes of Arunachal Pradesh and will support the development of comprehensive species management plans to safeguard these lesser-known flying squirrel species in the region. However, this study comes with some limitations for model evaluation, as the species had sporadic and limited presence sightings. Additionally, the probabilistic nature of the results means that changes in input variables could yield slightly different outcomes. Despite these constraints, the present study can serve as a foundation for further exploration and research initiatives, particularly when coupled with rigorous field validation, which will ultimately contribute to the conservation of these lesser-known species.

## Figures and Tables

**Table 1:** The table represents the model fit metrics for each of the participating modeling methods and for the final ensemble model for estimation of habitat suitability for the two flying squirrels in Arunachal Pradesh. A total of four model algorithms were used with the threshold of <0.75 AUC score. The models were Maximum Entropy (MaxEnt), Random Forest (RF), Generalized Linear Model (GLM), and Multivariate Adaptive Regression Splines (MARS). AUC: area under the curve, ΔAUC: change in the area under the curve (Training—Cross Validation), PCC: Proportion Correctly Classified, TSS: true skill statistic.

Species	Model	Dataset	AUC	ΔAUC	PCC	TSS	Kappa	Specificity	Sensitivity
*Petaurista mishmiensis*	GLM	Train	0.987	0.013	94.5	0.889	0.889	0.946	0.943
		CV	0.974		94.4	0.887	0.887	0.946	0.942
	MARS	Train	0.994	0.040	96.7	0.934	0.934	0.968	0.966
		CV	0.954		91.6	0.829	0.83	0.936	0.893
	MaxEnt	Train	0.974	0.032	95	0.9	0.9	0.946	0.955
		CV	0.942		92.7	0.854	0.853	0.924	0.929
	RF	Train	0.98	0.003	93.9	0.879	0.878	0.935	0.943
		CV	0.977		92.7	0.854	0.853	0.924	0.929
*Petaurista mechukaensis*	GLM	Train	0.976	0.076	92	0.842	0.833	0.917	0.925
		CV	0.9		85.2	0.708	0.693	0.844	0.864
	MARS	Train	0.963	0.035	89.7	0.794	0.785	0.898	0.896
		CV	0.928		84.1	0.671	0.664	0.862	0.81
	MaxEnt	Train	0.894	0.035	74.7	0.463	0.463	0.796	0.667
		CV	0.859		76.3	0.536	0.516	0.75	786
	RF	Train	0.92	0.012	83.4	0.669	0.656	0.833	0.836
		CV	0.932		85.2	0.698	0.686	0.872	0.826

**Table 2:** The mean percentage contribution of the covariates generated from the final model for the two flying squirrels in Arunachal Pradesh. Precipitation Seasonality: bio_15; Precipitation of Coldest Quarter: bio_19; Mean Diurnal Range: bio_2; Isothermality: bio_3; Temperature Annual Range: bio_7; Euclidean distance to River: euc_river; Human Influence Index: hum_foot; Montane Forests: montane.

Species	Variables	GLM	MARS	MAXENT	RF	μ (Mean)	μ (Mean) %
*Petaurista mishmiensis*	aspect	0	0	0.00194	0	0.000485	0.09
	bio_15	0	0.1965	0.10238	0	0.07472	13.18
	bio_19	0	0.43604	0.04734	0.00048	0.120965	21.34
	bio_2	0	0.04954	0	0	0.012385	2.19
	bio_3	0.18854	0	0	0	0.047135	8.32
	bio_7	0.20828	0	0.10346	0.01328	0.081255	14.34
	elevation	0	0.23594	0.03232	0	0.067065	11.83
	euc_river	0.1634	0.09014	0.08374	0.00398	0.085315	15.05
	hum_foot	0	0	0.00584	0	0.00146	0.26
	montane	0.15948	0	0.02264	0	0.04553	8.03
	slope	0.1142	0	0.0074	0	0.0304	5.36
*Petaurista mechukaensis*	aspect	0	0	0.001	0.001	0.0005	0.07
	bio_15	0.30726	0.02008	0.02498	0.0142	0.09163	13.46
	bio_19	0.34306	0.49132	0.32502	0.0004	0.28995	42.58
	bio_2	0	0.01118	0	0.00016	0.002835	0.42
	bio_3	0	0	0	0.00028	0.00007	0.01
	bio_7	0.29926	0.06036	0.10358	0.00008	0.11582	17.01
	elevation	0.15122	0.15754	0.10714	0.00012	0.104005	15.27
	euc_river	0.1771	0.0568	0.00352	0.0021	0.05988	8.79
	hum_foot	0	0	0.002	0.001	0.00075	0.11
	montane	0	0	0.05358	0.001	0.013645	2.00
	slope	0	0.00718	0.00002	0	0.0018	0.26

**Table 3:** The table represents the habitat quality and geometry of the suitable areas within the training area in present and future climatic scenarios for the two species. SSP: Shared Socioeconomic Pathways; NP: number of patches; PD: patch density; LPI: largest patch index; TE: total edge; LSI: landscape shape index; AI: aggregate index.

Species	Scenario	NP	PD	LPI	TE	LSI	AI
*Petaurista mishmiensis*	Present	253	37,376,848.5	7.5356	73.728	20.9234	81.7262
	SSP 245 (2041–2060)	243	35,899,502.7	5.4357	53.24	21.0629	78.9056
	SSP 245 (2061–2080)	219	32,353,872.8	2.8195	45.408	23.3214	71.9573
	SSP 585 (2041–2060)	238	32,513,416.7	3.1599	52.6	24.1074	70.92
	SSP 585 (2061–2080)	210	29,297,398	1.527	45.208	25.1094	66.2419
*Petaurista mechukaensis*	Present	76	11,227,828	4.5431	43.088	16.3576	81.0088
	SSP 245 (2041–2060)	98	14,477,988.7	4.2557	45.496	18.634	76.6144
	SSP 245 (2061–2080)	139	20,535,106.5	1.5591	32.776	17.2689	71.9054
	SSP 585 (2041–2060)	153	22,603,390.6	1.5828	48.928	20.0131	74.7021
	SSP 585 (2061–2080)	131	19,353,229.8	0.8339	30.936	17.6636	68.9848

## Data Availability

The data used in the analysis can be provided upon request to the corresponding authors.

## Supplementary Materials

The following supporting information can be downloaded at: https://www.mdpi.com/article/10.3390/biology14030242/s1, Figure S1. Figure showing the correlation between the covariates chosen for the final model for *Petaurista mishmiensis*. The Pearson correlation coefficient is primarily used here. However, if the Spearman or Kendall correlation coefficient exceeds the Pearson correlation coefficient, an “s” or “k” will be displayed in the bottom-right corner of the variable box. Figure S2. Figure showing the correlation between the covariates chosen for the final model for *Petaurista mechukaensis*. The Pearson correlation coefficient is primarily used here. However, if the Spearman or Kendall correlation coefficient exceeds the Pearson correlation coefficient, an “s” or “k” will be displayed in the bottom-right corner of the variable box. Figure S3. Evaluation matrix performance across model runs for *Petaurista mishmiensis*. Brown—represents the correlation coefficient among the four different models; Yellow—represents the proportion of deviance explained; Green—represents the Proportion Correctly Classified; Blue—represents the area under the curve (AUC); and Pink—represents true skill statistics. Figure S4. Evaluation matrix performance across model runs for *Petaurista mechukaensis*. Brown—represents the correlation coefficient among the four different models; Yellow—represents the proportion of deviance explained; Green—represents the Proportion Correctly Classified; Blue—represents the area under the curve (AUC); and Pink—represents true skill statistics. Table S1. The table shows the suitable area (in sq. km) across Arunachal Pradesh and the proportion of this area falling within the IUCN-designated range for the two flying squirrel species under present and future climatic scenarios.
