# Supplementary material for: Fading into Obscurity: Impact of Climate Change on Suitable Habitats for Two Lesser-Known Giant Flying Squirrels (Sciuridae: Petaurista) in Northeastern India
Source: Biology (Basel). 2025 Feb 27;14(3):242. doi: 10.3390/biology14030242 (PMC11940093; doi:10.3390/biology14030242)
Supplement: Supplementary file 1 [file biology-14-00242-s001.zip › biology-3473924-supplementary.pdf]

Supplementary Materials

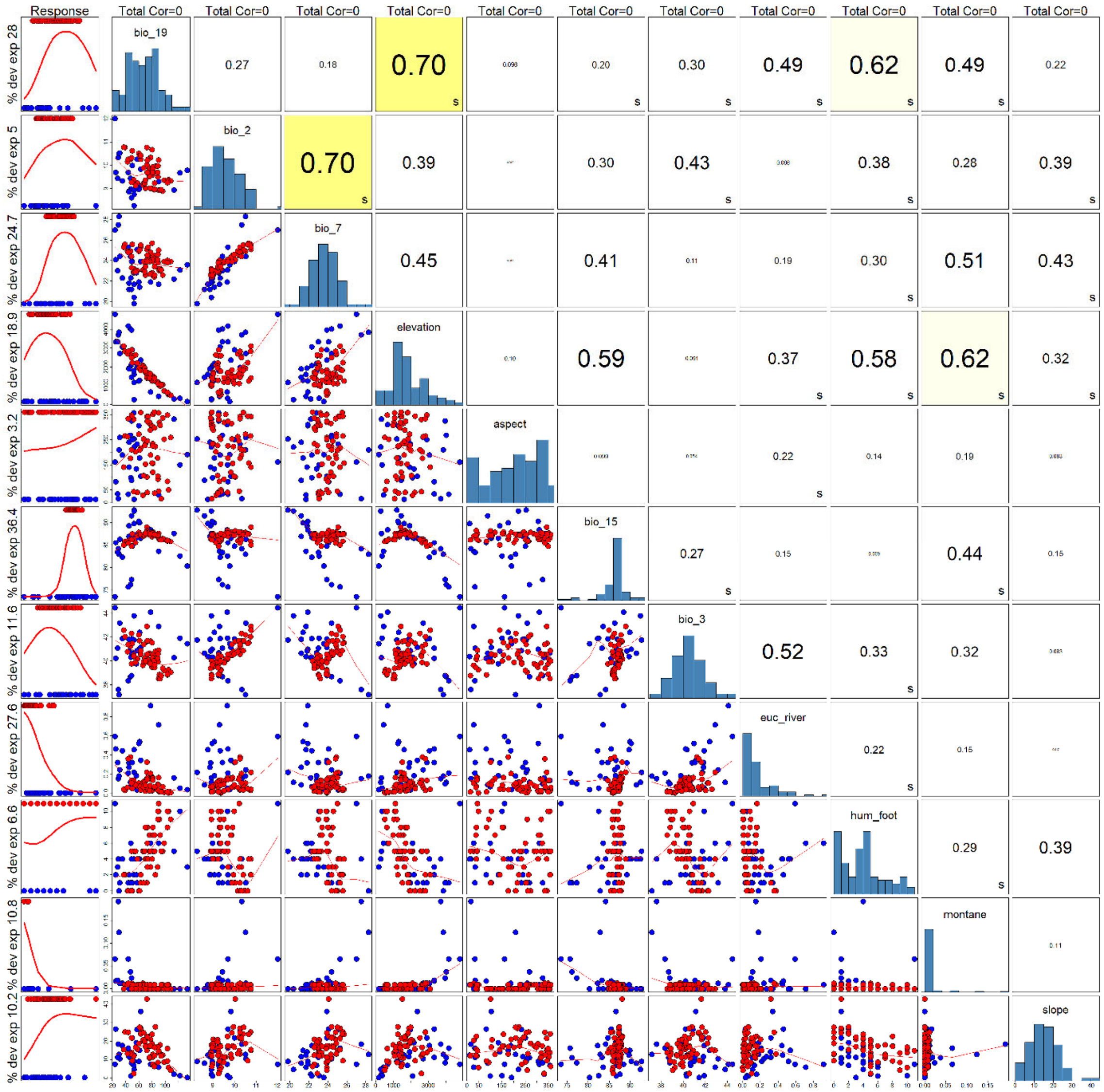

Figure S1. Figure showing the correlation between the covariates chosen for final model for *Petaurista mishmiensis*. The Pearson correlation coefficient is the primarily used here. However, if the Spearman or Kendall correlation coefficient exceeds the Pearson correlation coefficient, an "s" or "k" will be displayed in the bottom-right corner of the variable box.

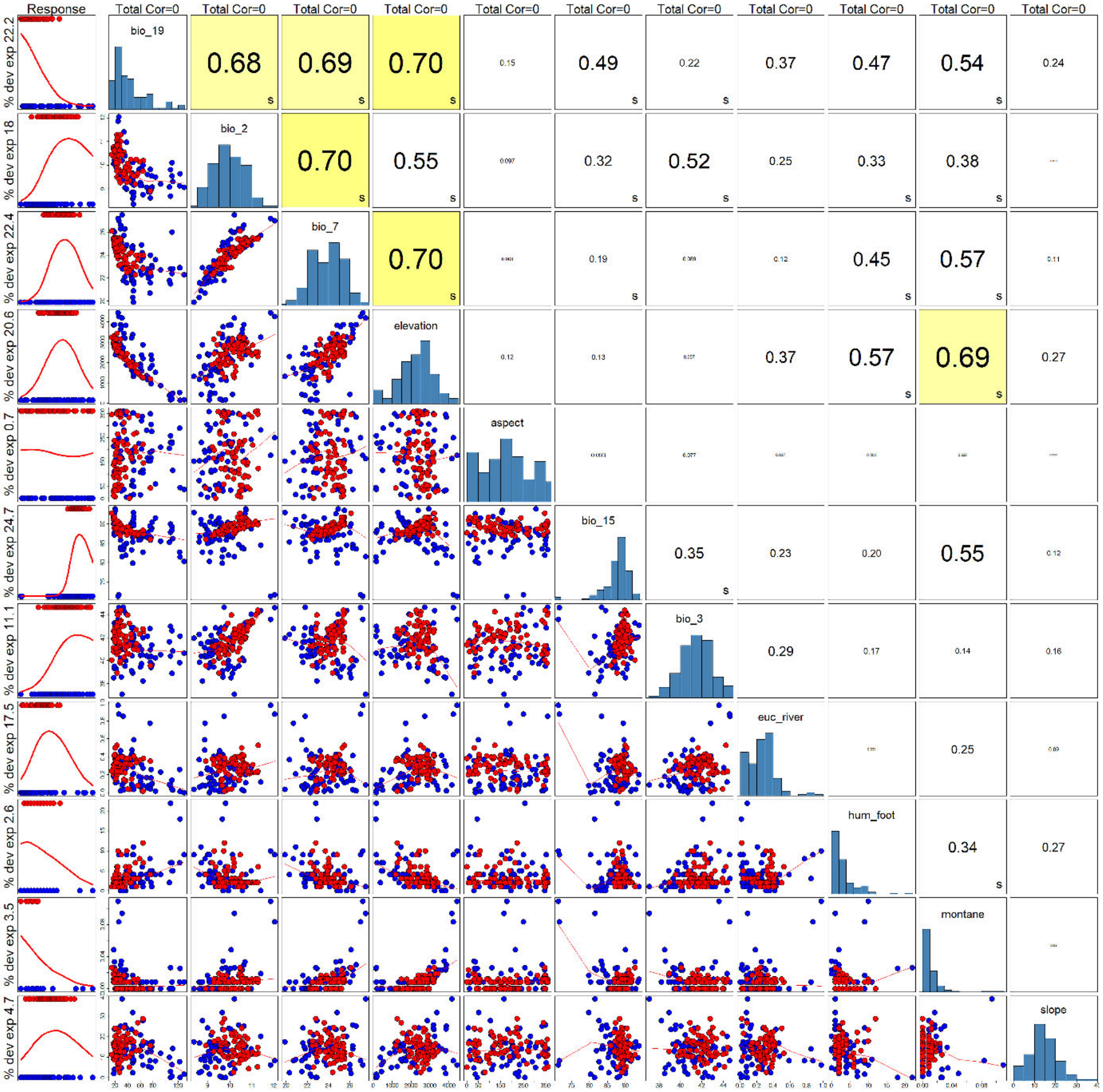

Figure S2. Figure showing the correlation between the covariates chosen for final model for *Petaurista mechukaensis*. The Pearson correlation coefficient is the primarily used here. However, if the Spearman or Kendall correlation coefficient exceeds the Pearson correlation coefficient, an "s" or "k" will be displayed in the bottom-right corner of the variable box.

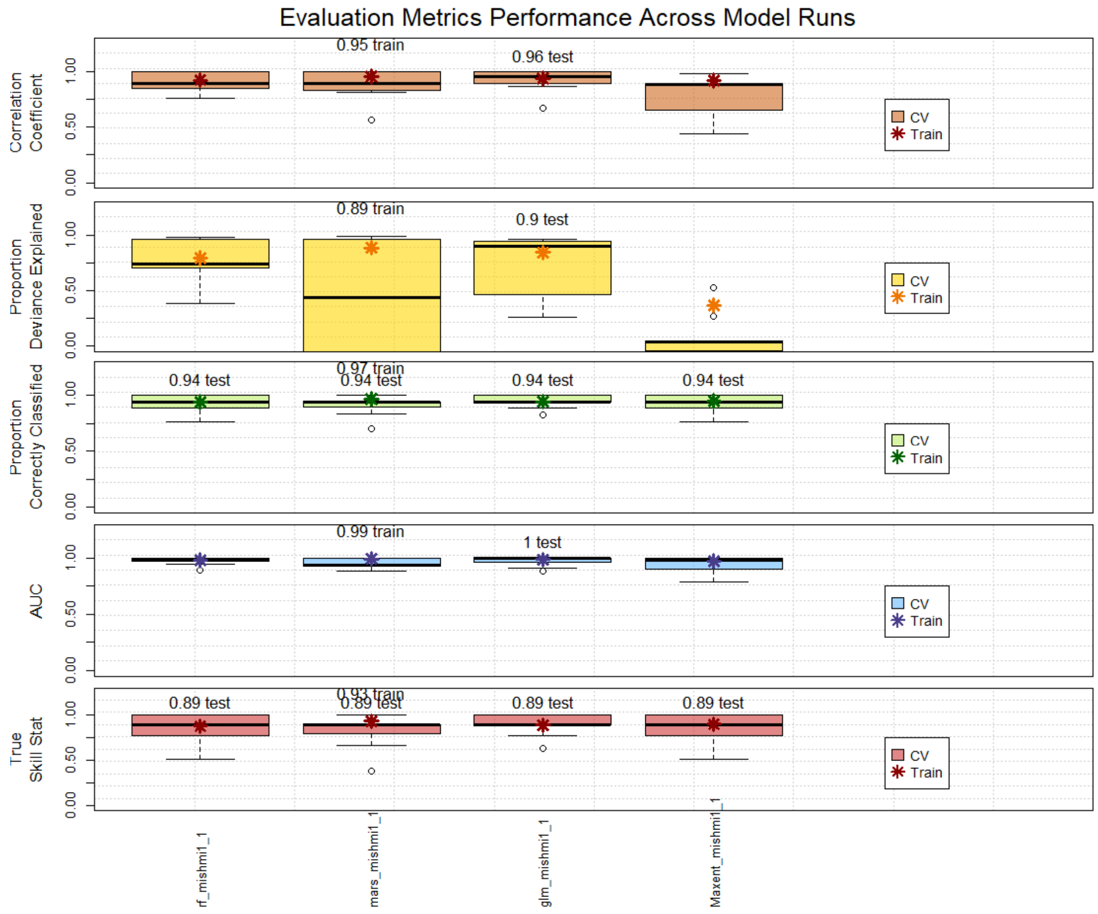

Figure S3. Evaluation Matrix performance across model runs for *Petaurista mishmiensis*. Brown - represents the correlation coefficient among the four different models. Yellow - represents the proportion of deviance explained; Green - represents the Proportion of correctly classified; Blue - represents Area under curve (AUC) and Pink - represents true skill statistics.

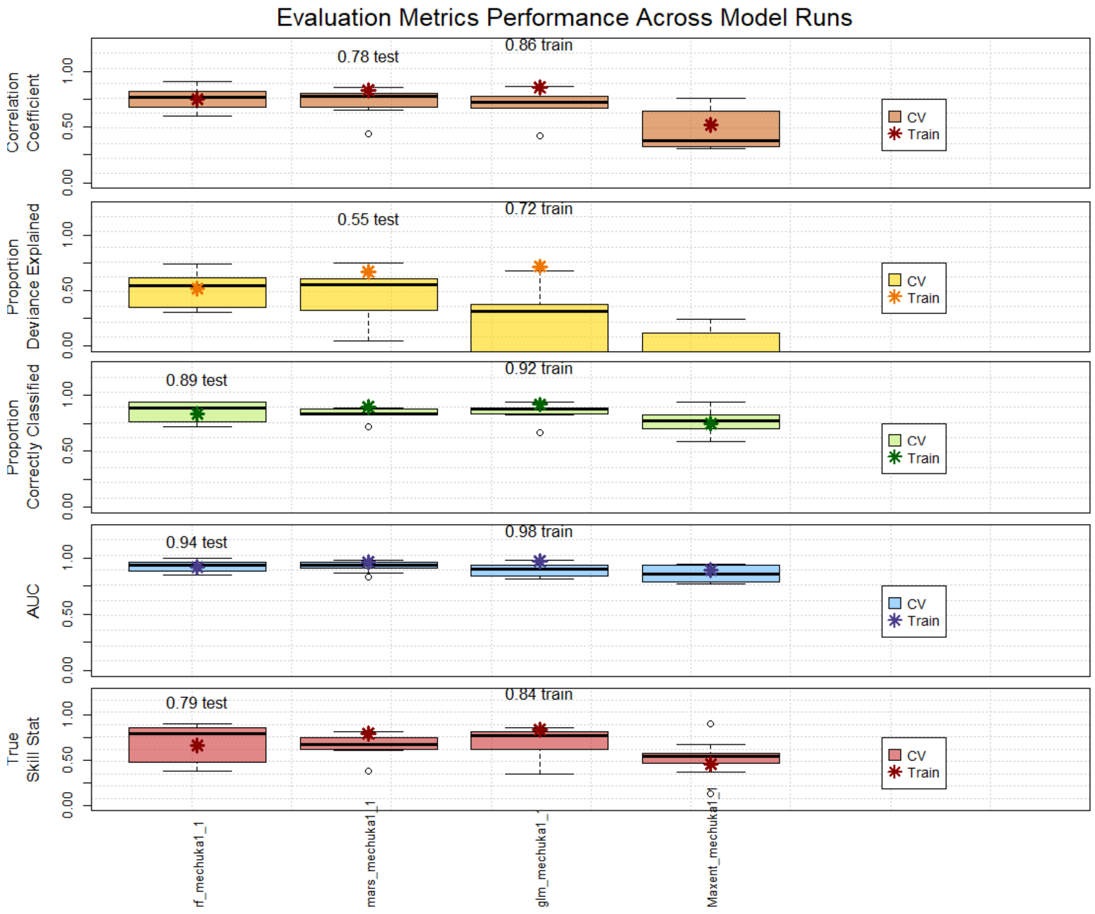

Figure S4. Evaluation Matrix performance across model runs for *Petaurista mechukaensis*. Brown - represents the correlation coefficient among the four different models. Yellow - represents the proportion of deviance explained; Green - represents the Proportion of correctly classified; Blue - represents Area under curve (AUC) and Pink - represents true skill statistics.

Table S1. The table shows the suitable area (in sq. km.) across Arunachal Pradesh and the proportion of this area falling within the IUCN-designated range for the two flying squirrel species under present and future climatic scenarios.

**Scenarios**	***Petaurista mishmiensis***		***Petaurista mechukaensis***	
	**Arunachal Pradesh**	**IUCN range**	**Arunachal Pradesh**	**IUCN range**
Present	9213	6917	6754	4571
SSP 245 (2041-2060)	7580	5343	5845	4278
SSP 245 (2061-2080)	4893	3909	3505	2888
SSP 585 (2041-2060)	5482	4013	5826	4410
SSP 585 (2061-2080)	4066	3122	3010	2161
